# A multi-site controlled trial of a cognitive skills program for mentally disordered offenders

**DOI:** 10.1186/1471-244X-12-44

**Published:** 2012-05-18

**Authors:** Angharad Rees-Jones, Gisli Gudjonsson, Susan Young

**Affiliations:** 1 University of Birmingham, Birmingham, UK; 2 King's College London, Institute of Psychiatry, London, UK

**Keywords:** Mentally disordered offenders, Treatment outcome, Reasoning & Rehabilitation, Cognitive skills

## Abstract

**Background:**

The effectiveness of offending behaviour programs in forensic mental health settings is not well established. Thus this study aimed to evaluate the Reasoning and Rehabilitation Mental Health program (R&R2 MHP) among a mentally disordered offender (MDO) population.

**Methods:**

A sample of 121 adult males drawn from 10 forensic mental health sites completed questionnaires at baseline and post-treatment to assess violent attitudes, locus of control, social problem-solving and anger. An informant measure of social and psychological functioning, including disruptive behaviour, was completed by unit staff at the same time. At three month follow-up patients completed again the violent attitudes and locus of control questionnaires. The data of 67 patients who participated in the group condition were compared with 54 waiting-list controls who received treatment as usual.

**Results:**

78% of group participants completed the program. In contrast to controls, significant treatment effects were found at outcome on self-reported measures of violent attitudes, rational problem-solving and anger cognitions. Improvements were endorsed by informant ratings of social and psychological functioning within the establishments. At follow-up significant treatment effects were found for both violent attitudes and locus of control.

**Conclusions:**

R&R2 MHP was effective in a sample of MDOs and had a comparatively low drop-out rate. Future research should use a randomized controlled design.

## Background

The number of people residing in secure hospitals and prisons is increasing [1] and reconviction rates suggest that within 5 years of release 15% of mentally disordered offenders (MDOs) will re-offend; 3% of whom will commit serious violent offences [2]. As prison and hospital populations are growing and the risk of re-offending remains, there is an increased demand for evidence-based treatments and rehabilitation strategies to alleviate this pressure. In the UK this has been amplified by the Ministry of Justice’s 2011 ‘payment by results’ directive [3] whereby in future organizations will be paid on the basis of outcomes achieved.

There is general agreement that criminal history, pro-criminal attitudes, associates and antisocial personality represent the “big four” risk factors [4] and it follows that these must be primary targets for change. Thus, a number of manualized programs have been developed that attempt to reduce the rates of reoffending through cognitive skills training [5] as research indicates that offenders either lack or have poor cognitive and social skills [6]. The most widely adopted programs have been the 36-session Reasoning & Rehabilitation program (R&R) [7,8] and 22-session Enhanced Thinking Skills (ETS) program [9]. R&R was the first manualized cognitive-skills program designed to specifically address antisocial and offending behaviour and accredited for use by the correctional services. It was developed by selecting cognitive techniques from programs that had been successful in reducing re-offending. It was designed to help offenders develop their cognitive and social skills and values and, thereby, improve their pro-social competence and decrease their reoffending. The major components of R&R are self-control, meta-cognition (thinking strategies as a means of regulating behaviour), social skills, interpersonal cognitive problem-solving skills, creative thinking, critical reasoning, social perspective-taking, values enhancement, emotional management and helper therapy (peer mentoring) [10,11]. Thus the program aims to modify cognitive skills and values; problem-solving skills are but one aspect and, although important, are secondary to the primary aim of pro-social competence. R&R has been widely researched; meta-analyses have supported its efficacy in a variety of settings with heterogeneous offenders and showing program attendees were 14% and 21% less likely to reoffend compared with controls when delivered in institutional and community settings respectively [12,13].

A growing interest has developed regarding the potential contribution that offending behaviour programs (OBPs) can make in treating MDOs. Whilst ETS and R&R were not designed to meet the complex needs of MDOs, uncontrolled pilot studies indicated they were effective in improving thinking styles and social problem-solving in this population [14-16]. Subsequent controlled studies of the R&R program have supported these findings [15,17,18] and a multi-site randomized controlled trial conducted by Cullen et al. [18] in medium secure services has reported specific improvements in social-problem solving post-treatment and at 12 month follow-up. However only half of those allocated to receive R&R completed the program; dropout was predicted by ‘high risk’ patients presenting with psychopathy, antisocial personality traits and violent behaviour [17]. This is a serious concern because non-completers have been reported to have higher rates of recidivism than completers or non-starters [19-21]. Thus, in order to maximize the benefits of rehabilitation programs a primary aim of treatment must be to promote and maintain engagement, and minimize program drop-out. One way to achieve this may be to combine group and individual work by the inclusion of a mentor whose role is to maintain engagement by supporting participants to consolidate the material introduced in the group and transfer acquired skills into daily activities [22,23]. Supplementation using a guided mentoring paradigm has been recognized by national guidelines in the treatment of antisocial personality disorder [24].

Secondly, the Responsivity Model [25] suggests that interventions should be tailored to specific offender characteristics, both in terms of content and pace, as opposed to delivering ‘broadbrush one-size fits all’ treatments. The Responsivity Model, first introduced in 1990 by Andrews, Bonta & Hoge [25], proposes three core principles of effective offender programming: (1) the ‘risk principle’ of directing services to prioritize higher risk offenders and minimizing services to low risk offenders, (2) the ‘need principle’ of targeting criminogenic needs in treatment and (3) the ‘responsivity principle’ requiring treatment to be provided in a style and mode that is responsive to the offender’s learning style and ability. Since then, the Responsivity Model has had a large impact on offender treatment policy by focusing attention on the need for structured and targeted treatment programs that aim to improve completion rates and reduce recidivism [3] and OBPs that adhere to the model have been shown to reduce offender recidivism by up to 35% [4].

The present study therefore aimed to evaluate a revised version of R&R that has been adapted to be responsive to the needs of MDO’s (R&R2 MHP) [26]. The program aims to maintain engagement through specific adaptations for a client group who commonly present with cognitive deficits (e.g. in attention and memory), and by including guided individual mentoring between group sessions. At 16 sessions, it is also much shorter than its 36-session predecessor. A small pilot study of R&R2 MHP delivered to offenders with severe mental illness in high and medium secure services reported a completion rate of 65%. Post-treatment per protocol analysis of 22 group completers compared with 10 waiting-list controls found improvement at outcome on measures related to self-reported violent attitudes and informant-reported disruptive behaviour. No significant improvements were found for social-problem solving or coping [27]. A similar R&R2 program, adapted for youths and adults with Attention Deficit Hyperactivity Disorder (R&R2 ADHD), piloted in 31 males with severe personality disorder detained in high security has also indicated good results with 76% of participants completing the program. Intention to treat analysis of 16 group participants compared with 15 waiting-list controls found improvement at outcome for self-reported social problem-solving, violent attitudes, ADHD symptoms, reaction to provocation (anger) and emotional control with mainly medium effect sizes [28].

This study aimed to evaluate the completion rate and effectiveness of R&R2 MHP using a waiting-list controlled design on a larger cohort of 121 MDOs detained in medium and low secure forensic facilities. Group attendees were compared with controls post-treatment on a primary outcome measure of violent attitudes and secondary outcome measures of locus of control, social problem-solving, reaction to provocation (anger), disruptive behaviour and social functioning. Measures of violent attitudes and locus of control were re-administered at three month follow up. It was hypothesized that group completion would be favorable compared with the 50% rate previously reported [17,18] and that group participants would show greater improvement at outcome than waiting-list controls.

## Methods

### Design and participants

This quasi-experimental controlled study involved the participation of 121 male patients detained under the UK Mental Health Act at 10 secure forensic facilities in the south-east of England (six medium secure and four low secure, N = 89 and N = 32 respectively). In order to optimize recruitment, patients from both low and medium security settings were invited to participate in the study. These settings differ in their staffing arrangements and physical security measures. Patients in medium security are those who present a serious danger to others and have the potential to abscond. Patients in low security are considered to present a less serious danger to others and security measures are intended to impede rather than prevent absconding. Usually patients go through an integrated care and treatment pathway that spans one or more levels of care.

All participants were referred by their clinical team to attend the group. Inclusion criteria for participants were (1) aged between 18–65, (2) had a current diagnosis or history of severe mental illness (e.g. schizophrenia, schizoaffective disorder, bipolar disorder), (3) a history of violent or antisocial behaviour leading to the current treatment episode, (4) not having participated in R&R or a similar program previously, (5) absence of learning disability and (6) proficiency in English language sufficient to allow participation in the program. Exclusion criteria included patients who were mentally unstable and/or who posed a risk of violence to the researcher.

The treatment group consisted of 67 patients who participated in the group condition (R&R2 MHP) and their data were compared with that of 54 waiting-list controls who received treatment as usual (TAU).

### Intervention

R&R2 MHP [26] consists of 16 90-minute sessions. It is a manualized CBT intervention program developed for antisocial youths and adults with mental health problems. It is a revised edition of the 36-session Reasoning & Rehabilitation program [8] that was originally developed as a pro-social competence training program for use in correctional facilities. R&R2 MHP is a structured, manualized program that aims to reduce antisocial attitudes and behaviour and improve cognitive and problem-solving skills. It consists of five treatment modules (1) neurocognitive, e.g. learning strategies to improve attentional control, memory, impulse control and constructive planning, (2) problem solving, e.g. developing skilled thinking, problem identification, consequential thinking, managing conflict and making choices, (3) emotional control, e.g. managing feelings of anger and anxiety, (4) pro-social skills, e.g. recognition of the thoughts and feeling of others, empathy, negotiation skills and conflict resolution, and (5) critical reasoning, e.g. evaluating options and effective behavioral skills. The program integrates group and individual treatment, the latter being achieved by the incorporation of a mentoring paradigm whereby a member of staff meets with the patient between group sessions to assist the participant to transfer skills learned in the group into their daily lives. Importantly the mentoring role is not devised to be an additive individual session; but aims to provide a structure for meetings or sessions that are routinely held between the participant and the designated staff mentor (e.g. primary nurse, keyworker, social supervisor). As a structured manualized program for both group facilitators and mentors, R&R2 MHP facilitates consistency in delivery and maximizes program integrity. All R&R2 MHP facilitators were experienced CBT practitioners and had received training in delivering the program. Mentors received written guidance about how to approach each mentoring session (included with program materials) and received training and onsite supervision from program facilitators. A steering committee was established in order to maintain a consistent approach to research and treatment and onsite supervision was carried out at each site. Thus treatment fidelity was ensured by the highly structured style of this manualized program, together with supervision provided at regular steering meetings by SY, an experienced clinical and forensic psychologist and program author.

#### Treatment completion

In line with the methodology applied by Cullen et al. [18], a cut-off equating to 80% of the program was applied to classify participants as completers (≥12 sessions) or non-completers (<12 sessions).

#### Treatment as usual

Participants were not asked to refrain from engaging in interventions considered to be part of their usual treatment with the exception that the control group were not permitted to attend R&R2 MHP sessions or other similar programd cognitive skills interventions such as R&R and ETS. Interventions that are commonly provided in medium and low secure settings include pharmacological treatments, individual and group occupational and psychological therapy, the latter including cognitive behavioral therapy for psychosis, anxiety, depression, substance misuse and relapse prevention.

### Measures

#### Baseline assessments

Demographic, diagnosis and index offence information was obtained from clinical file review at the start of the study. In addition participants completed the Patient Motivation Inventory (PMI) [29] to assess for possible variation in motivation to engage in treatment. This is a 16-item true/false questionnaire (score range 0–16). The PMI Total score has good internal consistency [30].

#### Outcome measures

The following measures were administered to assess the primary (violent attitudes) and secondary outcomes (locus of control, social problem-solving and reaction to provocation (anger), disruptive behaviour and social functioning). These measures are commonly used with mentally disordered offenders. All measures are self-rated with the exception of the Disruptive Behaviour and Social Problem Scale (DBSP) which is rated by an informant. All of the measures were administered at baseline (Time 1) and repeated post group (Time 2); measures relating to violent attitudes and locus of control were repeated at 3-month follow-up (Time 3).

1. Maudsley Violence Questionnaire (MVQ) [31] is a 56-item true/false questionnaire (score range 0–56) that measures cognitive style in relation to violent attitudes. The scale has two factors: machismo (endorsing stereotypical expectations of men as strong and tough) and acceptance of violence (enjoyment and acceptance of violence) (score ranges 0–42 and 0–14 respectively). The measure is reported to have high internal consistency (Cronbach’s alphas ranged from 0.76 to 0.91) in a male student sample and has specified differences between mentally disordered offenders [27].

2. The Locus of Control Scale (LoC) [32] was used to assess the extent to which participants believe events to be internally or externally controlled. It is a 40-item yes/no questionnaire with a high score indicating that the person perceives events as externally controlled whereas a low score indicates a person believes they control events internally (score range 0–40). The scale has been normed with depressed, psychiatric and low socio-economic populations and has been found to have adequate internal consistency (Cronbach’s alphas ranged from 0.37 to 0.86) [33]. Interventions should aim to increase a person’s internal orientation as research has found that people who have an internal locus of control (who perceive they are in control of life events) are more likely to participate in treatment and have more positive outcomes, whereas those with more external orientations (who believe life events are outside of their control and, for example, due to luck or fate) have been found to have poorer outcomes from treatment [34].

3. Social Problem-Solving Inventory Revised-Short Form (SPSI-RS) [35] is a 25-item questionnaire with responses rated on a 5-point Likert scale. The Inventory consists of five subscales, two measuring problem-solving orientation (positive and negative problem orientation) and three assessing problem-solving style (rational problem-solving, impulsivity/carelessness and avoidant) (scores range between 0–20 for each domain). An adjusted total score was obtained (score range 0–20) with higher scores reflecting better problem-solving ability. The measure is reported to have high test-retest reliability (Cronbach’s alphas ranged from 0.68 to 0.91) and internal consistency (Cronbach’s alphas ranged from 0.69 to 0.95), and positive correlations with other social problem-solving measures.

4. The Novaco Anger Scale and Provocation Inventory: Reaction to Provocation/Personal Affect Questionnaire (NAS-PI) [36] was used to assess cognitive, arousal and behavioral domains of anger experience. Forty-eight items on the scale, each rated on a 3-point Likert scale, provide these domains with higher scores indicating higher anger levels (scores range between 16–48 for each domain); a total score can also be obtained by summing the domain scores (score range 48–144). The NAS-PI has been shown to have good reliability (test-retest Cronbach’s alphas ranged from .78 to .91) and concurrent validity [37].

5. The Disruptive Behaviour and Social Problem Scale (DBSP) [38] in an informant-rated questionnaire consisting of 14 statements rated on a 7-point Likert scale relating to a person’s behaviour and social interactions (score range 14–98). The scale consists of two factors, (1) disruptive behaviour, e.g. whether the person is difficult to manage; if they are verbally aggressive or attention seeking (score range 8–56), and (2) social and psychological functioning, e.g. insight into behaviour, feelings of guilt, social interactions with others (score range 6–42). Higher scores indicate a greater degree of problems. Both factors have good internal consistency (Cronbach’s alpha 0.92 and 0.84 respectively).

### Procedure

Approval for the research was given by Ealing and West London Research Ethics Committee. Participants were referred by the clinical teams as meeting inclusion criteria and being suitable for the intervention. All patients at the facilities who were considered sufficiently mentally stable and who were ‘ready’ for this type of treatment and likely to benefit from it were referred by their clinical teams. The treatment was not mandatory. A waiting-list controlled design was applied in the study with group/control allocation being determined by the order of the referral. Once the number for a group had been reached, the remaining patients were put on a waiting list for the next group. After giving informed consent, participants completed the self-reported measures at baseline (Time 1) and data were extracted from the clinical records. A member of staff who knew the patient well (most commonly the primary nurse) completed the DBSP. To minimize between-rater differences, the same staff member was asked to complete the questionnaire at Time 1 and Time 2. Outcome measures were repeated again on completion of the group (Time 2) and MVQ and LoC were repeated three months later (Time 3). Only the primary outcome measure (MVQ) and the brief and relatively simple LoC measure were repeated at follow-up in order to reduce demand and maintain patient cooperation. It was not possible to collect follow-up informant data on the DBSP measure due to staff turnover on the wards. The timing of the assessments was generally the same for the R&R2M and TAU conditions. A total of 13 groups, each with 5–8 participants, were delivered running weekly. In addition group participants met with their mentor (an assistant or trainee psychologist) between sessions. Session logs were completed to record group attendance. All data was collected by researchers who were not involved in facilitating the groups. Information about other interventions was not collected and thus other treatments were not controlled for. Treatment integrity was ensured by the highly structured style of this manualized program (for both facilitators and mentors), regular attendance at steering groups by site representatives that included group discussion and supervision by SY a clinical and forensic psychologist and program author and, by arrangement, supplemental individual supervision sessions.

### Statistical analysis

**Figure 1:**
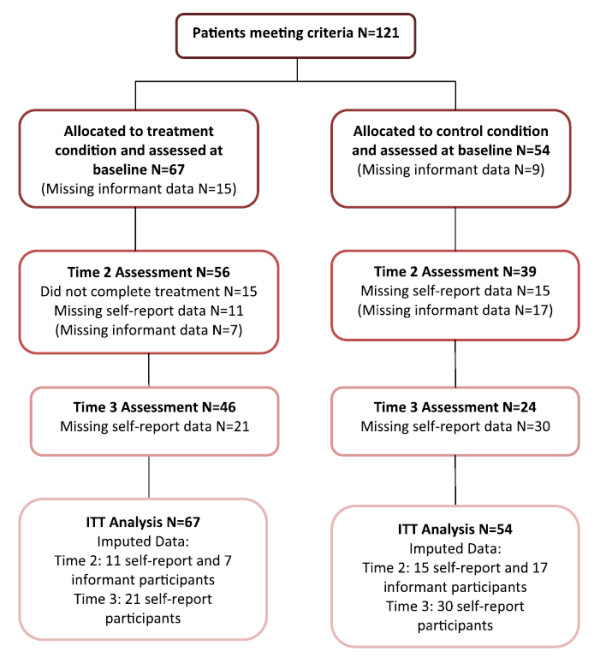
Flowchart of patient participation.

Descriptive statistics summarized demographics, clinical and forensic baseline characteristics. Independent-samples t tests were used to examine group differences at Time 1 (see Tables 1 and 2). Unadjusted mean scores and standard deviations on each of the outcome measures are provided in Table 3. All outcome analyses were intention to treat (ITT) and missing data were imputed by last observation carried forward (see Figure 1 for a flow chart of patient participation). Total score differences between the two conditions on the outcome measures were not statistically significant at baseline, nevertheless in order to minimize error variance an analysis of covariance (ANCOVA) was calculated for each of the dependent variables measuring differences between the conditions in time using adjusted mean scores and standard deviations. The baseline Time 1 scores therefore served as covariates for the dependent Time 2 and Time 3 variables. The effect size was analyzed using Cohen’s *d* for efficacy measures.

**Table 1:** Participant characteristics comparing group participants (R&R2M) and controls (TAU) at Time 1

	**R&R2M Group *N* M (SD)**		**TAU Group *N* M (SD)**		***χ***^**2**^**(df = 1)**
Security level	*67*	Medium = 42 Low = 25	*54*	Medium = 47 Low = 7	9.11**
					t-value
Mean age	*67*	34.14 (8.53) Range 19-62	*54*	35.56 (10.86) Range 20-65	-.80
Mean number of previous admissions ~	*54*	4.11 (3.75) Range 0-13	*48*	3.75 (4.56) Range 0-23	.44
Mean number of previous convictions ~	*58*	7.28 (13.47) Range = 0-93	*50*	8.96 (13.33) Range = 0-73	-.65
PMI Total Score	*67*	11.22 (3.31)	*54*	11.22 (3.55)	.003
MVQ Total Score	*67*	16.25 (12.61)	*54*	14.35 (11.28)	.86
SPSI-RS Total Score	*67*	11.70 (2.93)	*54*	12.61 (2.73)	−1.75
NAS-PI Total Score	*67*	82.43 (20.51)	*54*	76.93 (16.62)	1.60
LoC Score	*67*	16.13 (5.32)	*54*	116.04 (5.51)	.09
DBSP Total Score	*67*	37.23 (10.14)	*54*	37.89 (15.50)	-.25

~ = Data was not available from records for all participants.

** p < .01.

**Table 2:** Participant characteristics comparing group completers with non-completers at Time 1

	**Group completers *N* M (SD)**		**Non-completers *N* M (SD)**		***χ***^**2**^**(df = 1)**
Security Level	*52*	Medium = 33 Low = 19	*15*	Medium = 9 Low = 6	.06
					t-value
Mean Age	*52*	34.88 (8.77) Range = 20-62	*15*	31.60 (7.35) Range = 19-46	1.32
Mean number of previous admissions ~	*41*	3.78 (3.56) Range = 0-13	*12*	5.42 (4.34) Range = 0-12	−1.33
Mean number of previous convictions ~	*46*	6.95 (14.72) Range = 0-93	*11*	8.31 (9.16) Range = 0-30	−0.31
PMI Total Score	*52*	11.42 (3.14)	*15*	10.53 (3.89)	0.92
MVQ Total Score	*52*	16.46 (12.66)	*15*	15.53 (12.86)	0.25
SPSI-RS Total Score	*52*	11.30 (2.91)	*15*	13.09 (2.62)	−2.14*
NAS-PI Total Score	*52*	82.81 (21.27)	*15*	81.13 (18.25)	0.28
LoC Score	*52*	16.77 (5.42)	*15*	13.93 (4.43)	1.85
DBSP Total Score	*52*	37.48 (10.13)	*15*	40.40 (10.08)	−1.10

~ = Data was not available from records for all participants.

* p < .05.

**Table 3:** Post-treatment and follow-up ITT outcome data comparing R&R2M and TAU conditions

	**Baseline (Time 1)**		**Post-treatment (Time 2)**		**ITT Time 2 outcome**	**Follow-up (Time 3)**		**ITT Time 3 outcome†**
	R&R2M (N = 67) Mean (SD)	TAU (N = 54) Mean (SD)	R&R2M (N = 67) Mean (SD)	TAU (N = 54) Mean (SD)	F-value (Cohen’s d)	R&R2M (N = 67) Mean (SD)	TAU (N = 54) Mean (SD)	F-value (Cohen’s d)
MVQ Total Score	16.25 (12.61)	14.35 (11.28)	12.30 (10.10)	14.72 (10.43)	11.05 (.24) **	11.87 (10.06)	14.24 (10.70)	6.96 (.23) **
Machismo	9.73 (9.90)	8.17 (1.14)	6.48 (7.60)	8.41 (7.74)	11.23 (.25) **	6.24 (7.66)	8.17 (8.15)	6.62 (.24) **
Acceptance of Violence	6.52 (3.66)	6.19 (3.92)	5.82 (3.70)	6.31 (3.51)	3.80 (.14) *	5.63 (3.55)	6.07 (3.51)	3.18 (.13) *
LoC Total Score	16.13 (5.32)	16.04 (5.51)	15.76 (5.25)	15.88 (5.89)	0.06	14.78 (4.57)	15.90 (5.79)	3.49 (.23) *
SPSI-RS Total Score	11.70 (2.93)	12.61 (2.73)	12.55 (2.90)	12.84 (2.46)	0.37	-	-	-
Positive Problem Orientation	11.79 (4.25)	11.78 (4.09)	12.43 (4.22)	11.41 (4.40)	2.08	-	-	-
Negative Problem Orientation	7.39 (5.05)	6.83 (5.10)	7.10 (4.59)	6.28 (4.49)	0.64	-	-	-
Rational Problem Solving	10.36 (4.58)	10.81 (4.46)	12.00 (3.61)	10.63 (4.65)	6.21 (.33) **	-	-	-
Impulsivity/Carelessness	8.63 (5.16)	6.67 (4.07)	7.40 (4.81)	6.02 (3.95)	0.17	-	-	-
Avoidance Style	7.61 (4.64)	6.04 (4.25)	7.18 (4.13)	5.54 (4.08)	1.92	-	-	-
NAS-PI Total Score	82.43 (20.51)	76.93 (16.62)	77.42 (16.59)	76.81 (16.09)	2.58	-	-	-
Cognitive Domain	29.36 (5.99)	27.80 (5.51)	27.81 (5.09)	27.91 (5.64)	3.13 (.02) *	-	-	-
Arousal Domain	27.00 (7.66)	25.35 (6.41)	25.21 (6.36)	25.19 (6.23)	1.94	-	-	-
Behaviour Domain	26.07 (7.78)	23.78 (5.83)	24.40 (6.55)	23.72 (5.45)	1.17	-	-	-
DBSP Total Score††	37.23 (10.14)	37.89 (15.50)	35.60 (11.62)	39.07 (15.97)	2.78 (.25) *	-	-	-
Disruptive Behaviour††	16.27 (7.16)	16.89 (8.52)	16.12 (8.16)	17.06 (10.23)	0.55	-	-	-
Social and Psychological††	20.96 (6.64)	21.00 (9.25)	19.48 (6.72)	21.47 (8.89)	3.23 (.26) *	-	-	-

* p < .05, **p < .01 † Only MVQ and LoC measures administered at follow-up †† R&R2M N = 52; TAU N = 45.

In addition a post-hoc per protocol analysis was performed on the subgroup of participants for whom full data at Times 1, 2 and 3 were available. The analyses showed a similar pattern of results, thus only the ITT results are reported.

### Power calculation

Sample size calculations were based on data obtained in our pilot study [27]. Calculations performed at 80% power with an alpha level of 0.05 suggested that 35 participants per group were needed to detect a difference in the primary outcome measure of violent attitudes using the Total MVQ score with an effect size of .42 [pre-treatment mean 15.95 (S.D. = 10.83); post-treatment mean 11.36 (S.D. = 10.53)].

## Results

### Baseline characteristics

All participants had a current diagnosis or history of severe mental illness: psychotic disorders (N = 106; 87.6%), mood disorders (N = 15; 12.4%). All participants had a history of violent offending and for most patients this was the reason for the index offence N = 77 (63.6%) (e.g. homicide, sexual violence, use of firearms); other index offences for the current admission included: sexual (N = 20; 16.5%), financial (N = 11; 9.1%), arson (N = 7; 5.8%), drug-related (N = 5; 4.1%) and stalking (N = 1; 0.8%). Table 3 shows that R&R2 group participants comprised of 42 (62.7%) patients from medium security and 25 (37.3%) from low security; the TAU group comprised of 47 (87%) medium security patients and 7 (13%) low security patients. Hence, significantly fewer TAU participants were drawn from low security but there were no significant differences between R&R2 and TAU for age, previous number of admissions or convictions and PMI motivation to engage in treatment. No significant differences were found between the R&R2 and TAU groups on the total scores of the outcome measures administered pre-treatment (Time 1).

### Program completion rate

Table 2 shows that, of the 67 participants who commenced the group, 52 (78%) completed the group. Fifteen participants (22%) were classified as non-completers because they attended less than 12 sessions in total over the course of the program (n = 6), due to intentional withdrawal early in the program (n = 4), deterioration in mental state (n = 3), discharge (n = 1) or a clash in timetabling (n = 1). Group completers attended a mean of 15 (SD 1.12; range 12–16) sessions and the non-completers attended a mean of 7 (SD 3.36; range 0–11) sessions. There were no significant differences between completers and non-completers in age, previous convictions, previous admissions, and PMI motivation. At the beginning of treatment, the non-completers self-rated themselves on the SPSI-RS to have significantly better problem-solving skills than those who went on to complete the group (see Table 2); there were no other significant differences between the two groups on the total scores of the outcome measures administered pre-treatment (Time 1). There was no significant difference in the number of patients who dropped out from medium and low security (27% and 32% respectively; χ² = .06; df = 1; p = .81).

### Post-treatment outcome

Table 1 presents unadjusted means and standard deviations for each of the outcome measures at baseline and outcome (Time 2) for both R&R2 MHP and TAU. All effect sizes for significant results were small.

With respect to violent attitudes, R&R2 MHP scored significantly lower than TAU on the MVQ Machismo, Acceptance of Violence and Total scales. No significant differences were found between groups on the Locus of Control measure. For social problem-solving, the R&R2 MHP participants rated a significant improvement on the SPSI-RS Rational scale. No significant differences were found on the Total score or other subscales. Reactions to provocation (anger) were assessed by the NAS-PI. The R&R2 MHP participants rated a significant reduction in anger cognitions compared with TAU participants at outcome. There was no significant difference at outcome in the Total score or the Arousal and Behavior subscales.

An informant-report of functioning was assessed using the DBSP. R&R2 MHP participants were rated to show significant improvement on the Total score and Social and Psychological subscale compared with TAU participants. There was no significant difference between groups on the Disruptive Behaviour subscale.

### Outcome at follow-up

**Figure 2:**
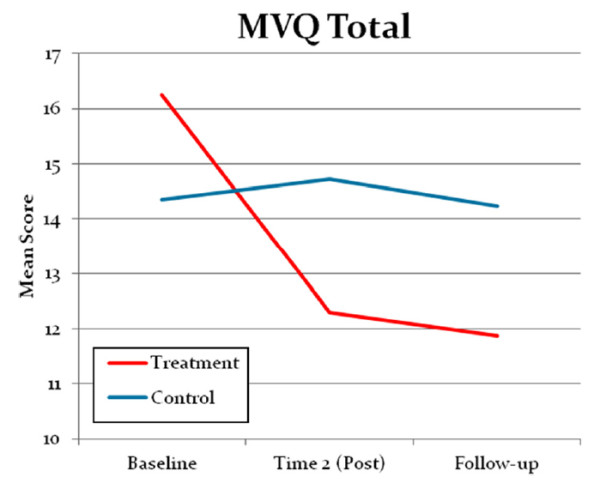
Self-reported changes in unadjusted means on the MVQ Total Score.

Two measures were re-administered at three month follow-up (Time 3), the MVQ and LoC. On this occasion the R&R2 MHP participants showed persistent significant improvement on the MVQ Total score (see Figure 2) Machismo and Acceptance of Violence subscales. Compared with TAU, R&R2 MHP participants had moved towards a more ‘normal’ locus of control at follow-up seen by the significant improvement in the LoC score.

## Discussion

This study aimed to evaluate the completion rate and effectiveness of R&R2 MHP which is a cognitive skills program developed for MDOs and derived from the Reasoning and Rehabilitation program. The program was initially piloted in medium and high secure settings [27] and the present findings support the feasibility of delivering the program to MDOs in medium and low security.

An important finding was the low drop-out rate, supporting the hypothesis that the group completion rate would be more favorable than that found in previously reported studies. The present study applied a very stringent completion rate of 80% attendance. The completion rate obtained in the present study of 78% is considerably higher than the rate of 50% reported by Cullen et al. [18] using the original 36-session version of the R&R program and applying the same completion criteria as the current study. Their sample was drawn solely from medium security whereas the current study included participants from low security, just over one-third of whom were in the treatment condition. As treatment drop out has been reported to be associated with risk status [17], it is possible that completion rates were inflated in the current study by the inclusion of lower risk patients who were more advanced in the rehabilitation pathway. Nevertheless comparison of group completers and non-completers in the present study showed no significant difference between groups in their motivation to engage in treatment, number of previous convictions and/or number of previous admissions to secure services, nor was there a significant difference in the number of patients who dropped out from medium and low security. Thus, as R&R2 MHP is 22 sessions shorter than its predecessor, program length and intensity of treatment may account for the favorable program retention.

A further and important influence on retention may be the specific adaptations that were made to the original program to improve responsivity. R&R2 MHP was designed to be more responsive to the needs of a forensic mental health population who are a more complex group of offenders, often presenting with severe mental illness, high rates of comorbid mental health problems, substance misuse and rigid cognitive styles. Moreover R&R2 MHP includes an individual mentoring paradigm which has been identified to be a supportive element associated with higher completion rates [22-24].

The association between non-completion of OBPs and recidivism is worrying; indeed it seems that it is better to not attend an OBP at all, than start one and drop out [19-21]. In the current study those who dropped out of treatment tended to have generally better social problem-solving skills, thus they may have perceived that a cognitive skills group was inappropriate and unlikely to meet their needs. However, the finding is inconsistent with that of a previous study reporting the reverse with poorer problem-solving skills being associated with program drop-out [39]. The present study did not obtain PCL-R scores which have been found to be an important marker of risk associated with drop-out [17]. It is a priority to identify predictors of treatment drop-out and develop methods to maintain engagement as this will have important implications for the selection of participants for group program and the management of offenders.

A second aim of the study was to evaluate the effectiveness of R&R2 MHP in MDO’s and, as hypothesised, significant treatment effects were found at outcome with small effects on self-reported measures of violent attitudes, rational problem-solving and anger cognitions. Improvements were endorsed by informant ratings of psychological and social functioning within the establishments. In order to reduce the load of the self-report battery only two measures were administered at follow up; one being the primary outcome measure of violent attitudes and the second, a relatively brief questionnaire, to determine locus of control. For the treatment group, significant small effects were found for these two measures at follow-up. Thus improvement was sustained over time suggesting that those who completed the intervention continued to use and consolidate the strategies learned in sessions after they finished treatment.

The present study found improvement on only one aspect of social problem-solving (i.e. rational) of the SPSI-RS. By contrast, other studies have reported post-treatment improvement in the Impulsivity/Carelessness, Avoidance and Total scales in MDO’s following treatment with the longer 36-session R&R intervention [17,18] and in offenders with severe personality disorder following treatment with the 15-session R&R2 ADHD [28]. At 12-month follow-up, Cullen et al. [18] found the effect for Impulsivity/Carelessness was sustained but results indicated less improvement in negative problem orientation compared with controls. As noted by Cullen et al., the R&R program may have differential impact on the varied functional modalities on the SPSI-RS with problem-solving orientation (positive/negative) being more resistant to change than problem-solving style. Cullen et al. [18] did not find a significant effect at outcome on the NAS-PI scales whereas the current study found a reduction in anger cognitions, possibly reflecting the greater focus on emotional monitoring and control strategies introduced in R&R2 MHP.

Nevertheless, in common with many multisite studies, a significant treatment effect was not found for every scale at outcome and, despite attempts to standardize the treatment and research protocols and ensure program integrity between the sites, there may have been variation in standards of delivery. Another possible explanation may be that most outcome measures were not re-administered at follow-up. The treatment effect at follow-up was sustained for violent attitudes and although there was no significant difference in locus of control between the two groups post-treatment, a small significant effect was present at follow up. Had other secondary outcome measures been repeated at follow-up, it is possible that a similar enhanced treatment effect may have extended to the SPSI-RS, NAS-PI and DBSP measures. This pattern of improvement has been reported in a randomized controlled trial of the R&R2 ADHD program delivered to outpatients with ADHD [40], emphasizing the importance of including follow-up evaluations to assess treatment outcome.

The results of the present study indicate that the R&R2 MHP program was effective in reducing antisocial thinking and behaviour, which is a primary aim of the program. Evaluation of R&R in correctional facilities has generally applied reconviction rates as the primary outcome measure [12,13]. Consistent with the findings of the present study, the ad hoc per protocol analysis of Cullen et al.’s [18] 12-month follow-up data found a treatment effect for violent attitudes. Thus ‘softer’ measures evaluating antisocial attitudes are likely to be important early markers due to their association with offending [41-44]. Thus antisocial attitudes and behaviors, together with reconviction rates, should be the primary benchmarks for evaluating OBPs in MDOs.

A strength of the study is the multi-site involvement, however participants were not randomly assigned to group condition. Thus in order to control for variance at baseline, ANCOVA was used with baseline Time 1 scores covarying for the dependent outcome scores and a more conservative ITT analysis selected over per protocol analysis. Nevertheless, high levels of staff turnover on wards meant that there were higher rates of missing informant data on the DBSP (that could be rated by the same member of staff across the two time points). One solution for future research would be to request that ratings are made collectively by the clinical team during ward rounds or clinical case conferences. Applying this method would additionally reduce informant bias. We also found that a record review was unhelpful as these were inconsistently recorded across sites; moreover critical incident records had a floor effect with most patients having no incidents recorded. Future research should consider using a prospective measure of aggression, such as the Staff Observation Aggression Scale Revised (SOAS-R) [45,46]. Multi-site trials are thus not without limitations due to within and between-site variations among procedures and participants. This ‘clustering’ of data is particularly salient to our inclusion of participants from low and medium security sites.

A second limitation was that the sample was exclusively adult males with severe mental illness and therefore the findings cannot be generalized to a wider offender population. Third, other characteristics may have influenced outcome that were not investigated, in particular IQ [39] self-esteem [47], impulsivity [48,49] and psychopathy [17] have been associated with non-completion rates. Fourth, four of the five outcome measures were self-report and we aimed to minimize a positive bias by these being administered by researchers who had not been involved in treatment provision. Fifth, clinical indicators were used to evaluate effectiveness and follow-up was relatively short. Nevertheless, despite these limitations, the data provide a good starting point for further development and investigation. Future randomized controlled evaluations need to be conducted to further reduce the potential for confounding variables, with a longer follow-up period, and objective measures including reconviction data. Program evaluation could be extended to offenders living in the community, females and those with learning disability to establish if this shorter program is responsive to the needs of these groups.

## Conclusions

The findings support the pilot study of R&R2MHP [27] and suggest that MDO inpatients from different levels of security are likely to benefit from a program that has been specifically adapted to take into account their level of functioning and clinical complexity. The primary aim of all of the R&R2 programs is to improve pro-social competence and this present study found this aspect significantly improved. R&R2MHP is considerably shorter than R&R and it is thus more resource and cost effective, in addition to being less labor intensive for both participants and facilitators. This is borne out by the considerably greater completion rates that are being consistently reported for the shorter R&R2 programs, the impact of which should not be underestimated in offenders given the association between completion rates, lower reconviction rates and ultimately public protection.

## Abbreviations

ADHD, Attention deficit hyperactivity disorder; DBSP, Disruptive behaviour and social problem scale; ETS, Enhanced thinking skills program; LoC, Locus of control; MDO, Mentally disordered offender; MVQ, Maudsley violence questionnaire; NAS-PI, Novaco anger scale and provocation inventory; OBP, Offending behaviour programs; PMI, Patient motivation inventory; R&R, Reasoning and rehabilitation; R&R2 MHP, Reasoning and rehabilitation 2 for adults with mental health problems; SPSI-RS, Social problem solving scale revised, short version; TAU, Treatment as usual.

## Competing interests

SY is a consultant for the Cognitive Centre of Canada and is co-author of R&R2MHP.

## Authors’ contributions

All authors contributed to the study design and management of the project. SY provided training in R&R2MHP. AR-J completed some of the data collection, conducted the statistical analysis and completed a first draft of the manuscript. AR-J, SY and GG made revisions and edits to subsequent drafts. All authors have read and approved the manuscript.

## Pre-publication history

The pre-publication history for this paper can be accessed here:

http://www.biomedcentral.com/1471-244X/12/44/prepub
